# Effects of sedentary behaviour and long-term regular Tai Chi exercise on dynamic stability control during gait initiation in older women

**DOI:** 10.3389/fbioe.2024.1353270

**Published:** 2024-05-09

**Authors:** Yuxia Chen, Chunxia Jin, Hongyuan Tang, Jinglun Yu, Yuanxin Wang, Shaolun Chen, Wensheng Miao, Shengnian Zhang, Xiangdong Wang

**Affiliations:** ^1^ School of Exercise and Health, Shanghai University of Sport, Shanghai, China; ^2^ Henan Sports Science and Technology Center (Henan Anti-Doping Center), Zhengzhou, Henan, China; ^3^ Huanghe Science and Technology College, Zhengzhou, Henan, China; ^4^ Henan Provincial Third People’s Hospital, Zhengzhou, Henan, China; ^5^ China Research Center on Aging, Beijing, Beijing, China; ^6^ School of Physical Education, Jimei University, Xiamen, Fujian, China

**Keywords:** Tai Chi, gait initiation, dynamic stability control, margin of stability, foot placement strategy, older women

## Abstract

**Background:** Sedentary behaviour has been associated with an increased risk of falls among older adults. Although gait initiation (GI) is a promising tool used to assess fall risk, it has yet to be quantitatively evaluated for dynamic stability in sedentary populations. Tai Chi exercise is believed to be effective in preventing falls in older adults, but its effect on GI stability has not been quantified. This study aims to compare the stability of GI in sedentary older individuals *versus* those who are long-term Tai Chi exercisers by using a quantitative approach.

**Methods:** This study included 17 sedentary older women without exercise habits (age: 65.59 ± 3.66 years, average daily sitting time: 8.735 ± 1.847 h/day) and 19 older women who regularly engage in Tai Chi exercise (age: 65.58 ± 3.63 years, years of exercise: 9.84 ± 3.48 years). Every participant underwent five trials of self-paced GI walking tests. Eight cameras and four force plates were used to obtain kinematic and kinetic parameters. The trajectory of the centre of mass (CoM) and the position of the foot placement were recorded. The anterior–posterior (A-P) and medio–lateral (M-L) dynamic stability at the onset and end moments of the single-legged support was calculated using CoM and gait spatiotemporal parameters. The stepping dynamic stability and foot placement positions of both groups were compared.

**Results:** The Tai Chi group had greater stability in the M-L directions at the swing leg’s toe-off moment and in the M-L and A-P directions at the heel-strike moment, as well as significantly larger step length, step width and step speed during locomotion than sedentary older women. However, the stability in the A-P directions at the swing leg’s toe-off moment and the foot inclination angle was not statistically different between the two groups.

**Conclusion:** Long-term regular Tai Chi exercise can enhance the dynamic stability of GI in older women, and effectively improve their foot placement strategy during GI. The findings further confirm the negative effect of sedentary on the stability control of older women and the positive role of Tai Chi in enhancing their gait stability and reducing the risk of falls.

## Background

Falling is an increasingly serious public health issue encountered by many aging countries. According to the fourth national health service survey conducted by the Ministry of Health in China, falls are the primary cause of injury (defined as undergoing medical treatment or having restricted activity for over a day after being injured) among key disease prevention and control measures. Falls account for the highest percentage (45.9%) of such injuries (Ministry of Health, 2008). The Centres for Disease Control and Prevention (CDC) in the United States issued a report (Centers for Disease Control and Prevention, 2023a), which highlights that falls are the principal cause of injuries and injury-related fatalities for persons aged 65 years and older. About one in four elderly people, or more than 14 million, experience a fall each year. Medical expenses incurred by such falls surpass $ 50 billion, with Medicare and Medicaid covering 75% of the cost (Kakara, 2023). The report indicates a higher proportion of falls amongst females (28.9%) than males (26.1%). Accidental falls caused the death of 38,742 elderly persons at a rate of 78.0 per 100,000 population in 2021. The mortality rate due to falls has been increasing, with a 30% increase over the past decade (Haddad et al., 2019; Centers for Disease Control and Prevention, 2023b).

The U.S. Preventive Services Task Force (USPSTF) recommends exercise interventions to prevent falls in community-dwelling adults 65 years or older who are at increased risk for falls (U.S.Preventive Services, 2018). Evidence-Based Falls Prevention Programs state that Tai Chi to be one of the most effective exercises for preventing falls (National Council on Aging, 2023). Studies have indicated a significant correlation among physical activity, reaction time and fall risk (Vala et al., 2023; Wang et al., 2023). Engaging in physical activity and avoiding prolonged sedentary behaviour can help reduce the risk of falls among older adults (Lee, 2020). In addition, dynamic stability of gait and lower limb support are considered important risk factors for falls among the older population (Yang and Liu, 2020). Community-dwelling older adults tend to experience falls most frequently during walking activities (Talbot et al., 2005).

With the advancement of sensor technology, wearable sensors of human daily activities provide significant opportunities for the development and evaluation of interventions aimed at improving the mobility of older adults (Boyer et al., 2023). For example, task-oriented movement learning programmes facilitated by sensor technology can enhance the mobility of older adults by improving the skills required for walking (Brach and VanSwearingen, 2013). In addition, neural networks and machine-learning approaches can help monitor human activity and detect and predict potential fall risks (Hartmann et al., 2023). Xue and Liu (2021) provided a comprehensive review of Hidden Markov Models, highlighting their considerable potential in handling sequential data and predicting hidden states, enabling the monitoring of human activities, gait and fall behaviour.

However, older adults are at a high risk of falling whilst performing common daily tasks, such as walking, changing posture or initiating steps in different environments (Talbot et al., 2005). However, there is currently a lack of simple and effective methods for predicting the risk of falls during gait initiation (GI). GI is one of the highest proportions of falls among older adults during motor activities (Robinovitch et al., 2013).

GI refers to the transition from a static standing posture to steady-state walking, which poses challenges to systems responsible for postural control (Winter 1995). GI necessitates the transition from standing to a stable gait, so it provides an ideal task for studying the mechanisms of falls in older adults and the effect of ageing on mobility (Muir et al., 2014). Evaluating the stability of GI in sedentary older adults, who engage in activities, such as sitting, lying down or reclining with energy expenditure ≤1.5 metabolic equivalents while awake (Atkin et al., 2012; Edwardson et al., 2012), may serve as a valuable tool for assessing fall risk.

Tai Chi exercise is an effective intervention for reducing falls among older adults (Chen et al., 2021; National Council on Aging, 2023). Tai Chi movements require slow, continuous and smooth actions, which involve the continuous transformation of weight-bearing and non-weight-bearing movements and maintaining body stability. Various studies have highlighted the positive effects of Tai Chi on balance control and fall prevention in older adults (Chen et al., 2023). The present study aims to quantitatively assess the stability of GI in sedentary older adults and long-term Tai Chi exercisers by using this functional task to determine the risk level of sedentary individuals and the effectiveness of Tai Chi intervention.

Voluntary movement of GI is a means to accomplish stable walking tasks. The human body constantly adapts to natural instability triggered by variations in the supporting surface during movement. The central nervous system (CNS) employs stable and effective mechanisms to handle instability inherent in GI. The effectiveness of executing GI tasks, which refers to the output efficiency of sensory–motor system integration, is typically evaluated based on movement performance during GI, such as speed and accuracy (Bouisset and Do, 2008). The process of GI involves two skills, namely, forward propulsion and balance control, which are accompanied by posture–locomotion coupling (Mille et al., 2014). Successful initiation of gait requires two biomechanical requirements, which are generation of momentum (forward direction and towards the supporting leg) and maintenance of balance (Polcyn et al., 1998). Lifting the swing leg and taking a step forward can result in a potential sideway imbalance of the body, which can be effectively stabilised by the CNS. This task is accomplished by shifting the centre of pressure (CoP) of the anticipatory postural adjustments (APAs) towards the swing leg, moving the centre of mass (CoM) towards the supporting leg and partly through effective stepping (Winter 1995).

A few definitive quantitative studies have been conducted on the stability of GI in sedentary individuals and Tai Chi exercisers. The most commonly used indicator for quantifying dynamic stability during GI is the concept of ‘margin of stability (MoS)’ proposed by Hof et al. (Hof et al., 2005). MoS is a composite variable that considers the relationship among CoM position, velocity and support surface and has been widely applied to quantify stability during GI tasks. MoS is a measure of walking stability derived from dynamic stability theory and the human inverted pendulum model; it represents the minimum distance from a given CoM position–velocity state point to the backward imbalance boundary. Stability during GI is evaluated by calculating MoS in the medial–lateral (M-L) and anterior–posterior (A-P) directions, with the M-L side being considered a key indicator of walking stability.

This study utilises Hof’s gait stability assessment method (Hof et al., 2005; Hof, 2008) to collect biomechanical data on the kinematics and kinetics involved in the GI of older participants. The method calculates the stability of M-L and A-P directions during the double-support to single-support transition of the swing leg’s toe-off as well as at the end of the swing leg heel–strike when shifting from single to double support. The study compares the dynamic stability of sedentary and long-term regular Tai Chi practising older females during voluntary GI and evaluates the foot placement characteristics at the end of GI to explore the potential fall risk for sedentary individuals and the effectiveness of Tai Chi in preventing falls. Based on research findings, older women may be more susceptible to falls than older men (Stevens and Sogolow, 2005; Johansson et al., 2016; Yogi et al., 2018; Peng et al., 2019; Moreland et al., 2020). To minimise gender bias, this study exclusively focused on female participants. The research aims to: (1) determine differences in the dynamic stability of GI between sedentary individuals and those who engage in regular long-term Tai Chi exercise; and (2) compare foot placement strategies during stepping. The hypotheses are as follows: (1) sedentary individuals are expected to present significantly lower dynamic stability of GI than Tai Chi exercisers; and (2) both groups are assumed to exhibit distinct foot placement strategies. Results will provide evidence regarding factors contributing to fall risk in older individuals and the effects of exercise interventions.

## Methods

### Study sample

The sample size was estimated using G*power software, which determined that an effect size of 0.5 and a statistical power of 80% (*α* = 0.05) are necessary to detect a significant difference (Zhang et al., 2002), requiring at least 51 trials in each group. The study finally recruited 128 elderly participants, with ineligible individuals, dropouts and invalid data excluded, included 36 participants, each of them completed five trials, resulting in 166 trials, which satisfied the requirement for statistical significance.

### Participants

The inclusion criteria for the participants were as follows: female, right-leg dominance; good physical health; absence of back and pelvic system issues, neuromuscular disorders or balance impairments; and normal cognitive function. The sedentary group (SG) was defined as those who reported no exercise habits and an average sedentary time greater than 6 h per day in the past 7 days, and the Tai Chi group (TC) had more than 6 years of training.

Participants with a Mini-mental State Examination (MMSE) functional score of 24, frailty, lower limb injuries or surgeries within the past 6 months, a history of falls in the previous 2 years and fatigue or poor condition prior to the experiment were excluded.

All participants were informed about the specific test procedures and signed written informed consent forms. The study was approved by Henan Sports Science and Technology Centre (Henan Anti-Doping Centre), China.

### Instrumentations

Kinematic data were obtained using eight 3D near-infrared high-speed cameras (Qualisys, Sweden, model: Oqus 600) with a sampling frequency of 100 Hz. Fifty-two infrared reflective marker points (diameter of 14 mm) were attached to the participants to collect GI kinematic information. The placement method of the markers follows the Helen Hayes model. Kinetic testing was conducted using four Kistler force plates (90 cm × 60 cm ×10 cm, Switzerland, model: 9287C), with a sampling frequency of 1,000 Hz. A remote-controlled start signal lamp was self-constructed and used.

### Experimental procedures

Participants were instructed to refrain from engaging in strenuous physical activities for 3 days prior to the experiment and to avoid consuming stimulating beverages, such as alcohol or coffee, which might affect the nervous system on the day before the experiment. Sufficient sleep was ensured. On the day of formal testing, participants were required to familiarize themselves with the experimental procedures and exercise them thoroughly. After the experimental preparations were completed, the participants performed a 5-min warm-up activity before proceeding to the formal testing. The starting action involved participants standing on a force plate with standardised footwear and clothing, with each foot placed on a force plate. They were instructed to stand naturally in a static position with their feet shoulder-width apart. The signal indicator is located at the endpoint, which is opposite to the walking direction of the subject 1.2 m above the horizontal plane and 6 m away from the starting position of GI.

The GI walking test was performed under the experimental condition designed in this study. The participants stood naturally on the force plate for 3–5 s and then initiated a step forward voluntarily. Trials started with the subject in quiet standing, with their feet placed in their preferred natural stance. Following a verbal cue of ‘anytime’, the subjects waited for a self-selected time interval (no less than 3 s) then initiated forward stepping at their self-chosen pace. Five valid trials were collected and reviewed online to ensure a steady-state baseline before the ‘anytime’ instruction. Trials without a period of steady-state standing were discarded and repeated.

Data of gait evaluation were analysed using Visual3D software (C-Motion, USA, v6.01.36). Kinematic and kinetic data were filtered and denoised by using a lowpass filter, with cut-off frequencies of 6 Hz for kinematics and 20 Hz for kinetics.

The GI process is divided into two phases: the APA phase and the locomotion (LOC) phase. This study focused on analysing the stability of the LOC phase. The MoS at the onset of LOC as well as the forward and lateral control stability at the end of LOC were computed.

## Dynamic Stability of Gait Initiation

### Margin of stability at the onset stepping of locomotion phase

Next to the position and the velocity of the CoM, we can introduce the ‘extrapolated centre of mass (XcoM)’, as shown in Eqs 1, 2, where ω_0_ is a constant related to stature. XcoM allows the requirements for stable walking to be formulated simply based on the inverted pendulum model of balance.

$$\omega_{0}=\sqrt{g/l}$$ (1)

$$\text{XcoM}=\text{CoM}+\frac{V_{CoM}}{\omega_{0}}$$ (2)

Where XcoM represents the XcoM position, $V_{CoM}$ is the CoM velocity and normalised by the eigenfrequency $\omega_{0}$ , l is the leg length measured from the lateral malleolus to the greater trochanter and g represents the gravity acceleration (Hof et al., 2005).

The minimum M-L margin of stability MoS_ML_ and A-P margin of stability MoS_AP_ were computed as follows in Eqs 3, 4:

$${\text{MoS}}_{\text{ML}}=\text{BoS}\left(x\right)-\text{XcoM}\left(x\right)$$ (3)

$${\text{MoS}}_{\text{AP}}=\text{BoS}\left(y\right)-\text{XcoM}\left(y\right)$$ (4)

**FIGURE 1:**
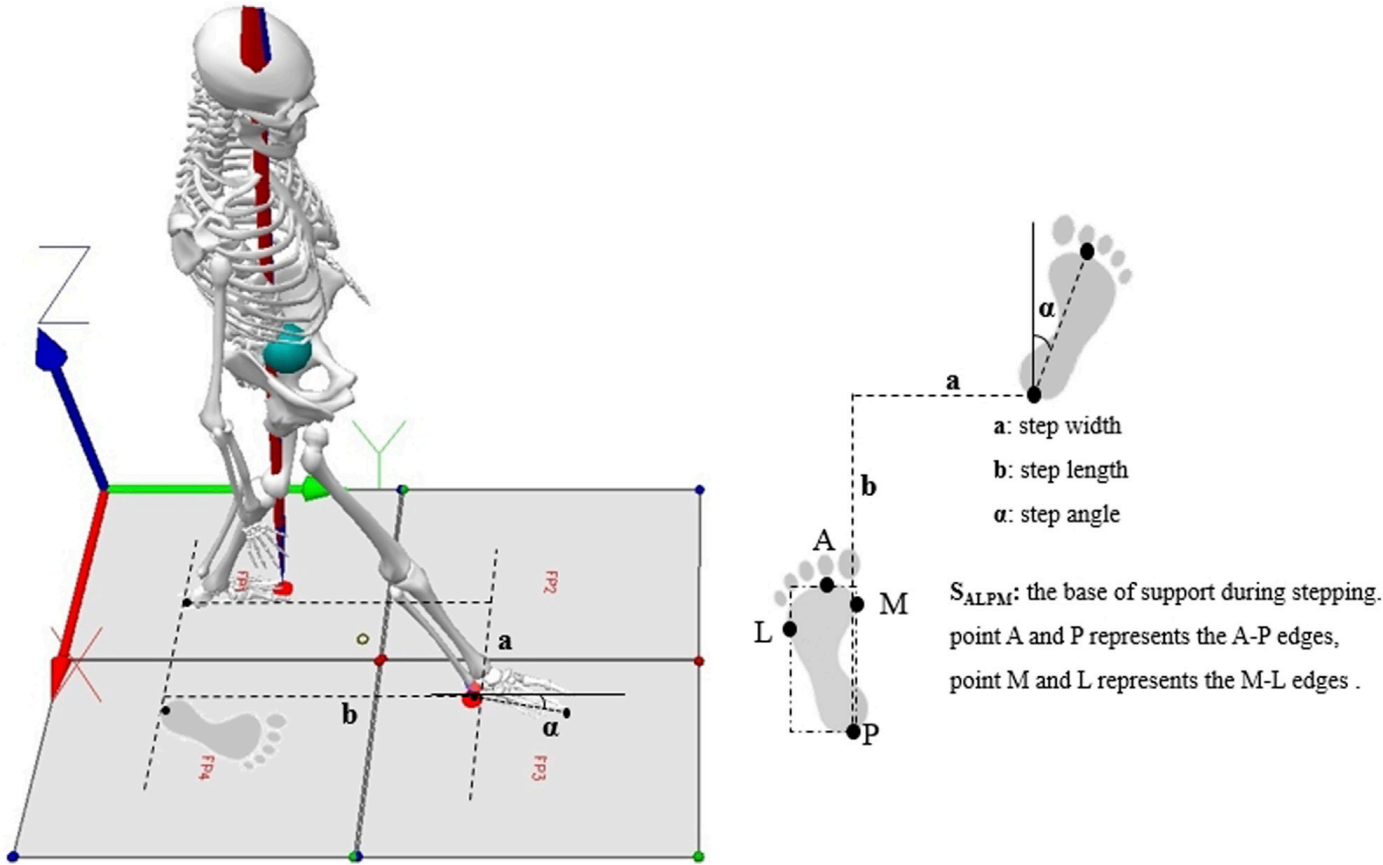
Base of support and position of foot placement.

MoS_ML_ and MoS_AP_ were calculated as the distance between XcoM and the boundaries of the base of support (BoS) during the single stance from the foot markers, namely, the fifth metatarsal–phalangeal joint for the lateral border and the medial malleolus for the medial border (Hof et al., 2005; Osada et al., 2022), as illustrated in Figure 1.

## Stability control at the end of the locomotion phase

### Lateral stability control

We calculated lateral stability with a constant offset control by positioning the right foot B_ML_ to the right of the XcoM, which is related to the desired step width SW in Eq. 5 (Hof, 2008).

$$B_{ML}=\frac{SW}{e^{\omega_{0}t}+1}$$ (5)

Where $B_{ML}$ is the stability of lateral control, SW is the step width and t is the time of LOC.

### Forward stability control

The simplest stable control of CoP position could be made by positioning the CoP at a constant distance behind the ‘constant offset control’ of XcoM. The offset value B_AP_ is represented as a constant distance. The equation is as follows in Eq. 6 (Hof, 2008).

$$B_{AP}=\frac{SL}{e^{\omega_{0}t}-1}$$ (6)

Where $B_{AP}$ is the stability of forward control, SL is the step length and t is the time of LOC.

### Foot placement at the end of stepping

Foot placement was determined based on step length, step width and foot inclination angle, as illustrated in Figure 1.

### Statistical analysis

Statistical analysis was performed using SPSS software (version 22, SPSS Inc., Chicago, IL USA). Shapiro–Wilk test was used to verify the normal distribution of parameters; if the parameters were normally distributed, the Independent Samples *t*-test was applied to compare data between SG and TC groups, if the parameters were not normally distributed, the Mann–Whitney *U* test was applied. Effects were considered to be significant at *p* < 0.05.

## Results

**FIGURE 2:**
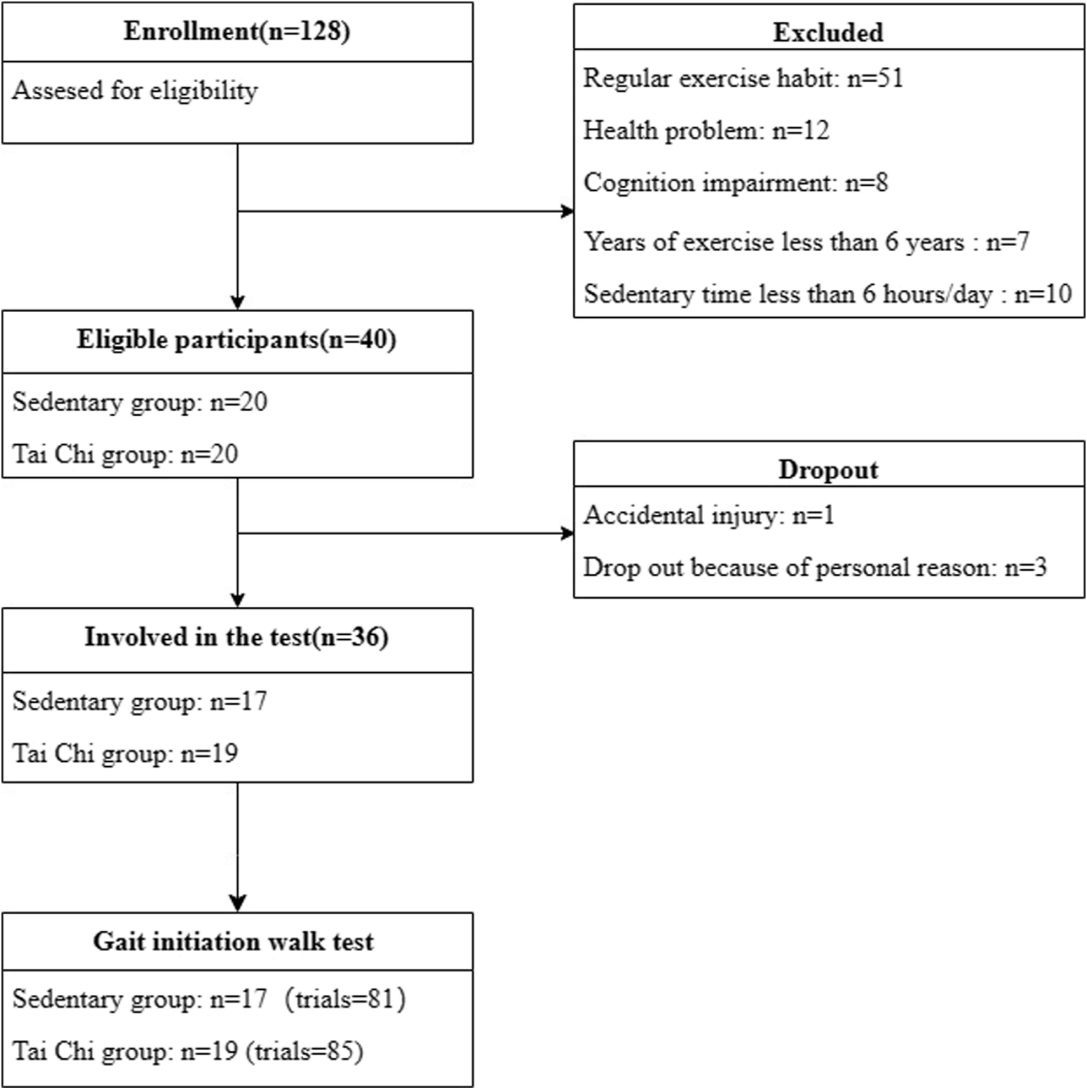
Recruitment of participants in sedentary and Tai Chi groups.

The recruitment process for participants of both surveys is illustrated in Figure 2.

### Sample characteristics

The study included 17 older females who did not engage in regular exercise (age: 65.59 ± 3.66 years; height: 1.62 ± 0.53 m; mass: 65.41 ± 12.25 kg; BMI: 26.04 ± 7.79; MMSE scores: 28.7 ± 0.40) and 19 older females who participated in long-term Tai Chi exercise (age: 65.58 ± 3.63 years; height: 1.58 ± 0.43 m; mass: 60.94 ± 7.27 kg; BMI: 24.32 ± 2.72; MMSE scores: 28.9 ± 0.49). Table 1 provides detailed information about these individuals.

**TABLE 1:** Information of participants in sedentary and Tai Chi groups.

	SG	TC	*p*-value
	Mean ± SD	Mean ± SD	
*n* (trials)	17/(81)	19/(85)	
Age (years)	65.588 ± 3.658	65.579 ± 3.626	0.994
Height (m)	1.615 ± 0.0530	1.584 ± 0.043	0.062
Mass (kg)	65.406 ± 12.251	60.937 ± 7.273	0.187
BMI (kg/m^2^)	26.039 ± 3.788	24.324 ± 2.719	0.125
MMSE (scores)	28.645 ± 0.400	60.937 ± 7.273	0.083
Sedentary time (hours/day)	8.735 ± 1.847	3.526 ± 0.456	<**0.001**
Physical activity frequency (times/week)	—	5.947 ± 1.471	
Physical activities duration (hours/day)	—	1.526 ± 0.390	
Years of exercise (years)	—	9.842 ± 3.480	

SG: sedentary group; TC: long-term regular Tai Chi exercise group.

### Dynamic stability

Table 2 indicates statistically significant differences in lateral stability between the SG and TC groups at the beginning of the single-support phase during the transition from double to single support during GI. The MoS_ML_ result in SG is significantly lower than that in the TC group (*p* = 0.19, η^**2**^ = 0.342, 95%CI: −0.011, −0.001). M-L and A-P stability also show statistically significant differences at the end of GI when the swing leg touches the ground and shifts from single to double support. The B_ML_ (*p* = 0.002, η^**2**^ = 0.455, 95%CI: −0.013, −0.003) and B_AP_ (*p* = 0.001, η^**2**^ = 0.473, 95%CI: −0.009, −0.002) results in SG are significantly lower than those in the TC group.

**TABLE 2:** Dynamic stability of sedentary and Tai Chi groups on the onset and end of locomotion.

	SG	TC	*p*-value	η^2^	95% CI	
	Mean ± SD	Mean ± SD			CI_L_	CI_U_
MoS_ML_ (m)	−0.028 ± 0.007	−0.022 ± 0.010	**0.019**	0.342	−0.011	−0.001
MoS_AP_ (m)	−0.068 ± 0.033	−0.074 ± 0.031	0.506	0.399	−0.013	0.025
B_ML_ (m)	0.040 ± 0.007	0.048 ± 0.009	**0.002**	0.455	−0.013	−0.003
B_AP_ (m)	0.022 ± 0.002	0.028 ± 0.008	**0.001**	0.473	−0.009	−0.002

SG: sedentary group; TC: long-term regular Tai Chi exercise group.

### Foot placement

Table 3 shows that the TC group has a greater step length, step width and step speed than SG at the end of the GI process when the swing leg heel makes contact with the ground and transitions from single to double support. The statistical analysis reveals significant differences between the two groups, with a more pronounced difference observed in SL and SW. The foot inclination angle (SG: 33.097 ± 29.219; TC: 31.333 ± 20.671, *p* = 0.821, η^**2**^ = 0.352, 95% CI: 13.893, 17.42) is higher in SG than in the TC group.

**TABLE 3:** Foot placement in sedentary and Tai Chi groups at the end of gait initiation.

	SG	TC	*p*-value	η^2^	95% CI	
	Mean ± SD	Mean ± SD			CI_L_	CI_U_
SL(m)	0.545 ± 0.060	0.595 ± 0.045	<**0.001**	0.439	−0.071	−0.029
SW(m)	0.176 ± 0.030	0.206 ± 0.039	**0.006**	0.403	−0.051	−0.009
SV(m/s)	0.155 ± 0.055	0.307 ± 0.268	**0.016**	0.476	−0.273	−0.031
SA (°)	33.097 ± 29.219	31.333 ± 20.671	0.821	0.352	−13.893	17.420

SG: sedentary group; TC: long-term regular Tai Chi exercise group.

## Discussion

GI is a voluntary movement task that involves the transition from standing to walking. During GI, the CNS continuously compares the expected movement with the actual movement in the environment, modifying the control of movement to identify the most effective and efficient method to achieve the goal (Hadders-Algra, 2010). The stability of the body’s control changes due to changes in the support surface during GI, and the CNS uses stable and effective mechanisms to deal with the inherent instability of GI. The function of the CNS during GI is to shift the CoM in the desired direction and to determine the placement of the swing foot upon landing (Winter 1995). The efficiency of GI performance, which refers to the output efficiency of sensory–motor system integration, is typically evaluated based on GI movement characteristics, such as speed and accuracy. The present study aims to assess differences in GI performance efficiency between sedentary older women and those who have undergone long-term Tai Chi training by assessing stability at the moment of transition from double support to single support during swing leg initiation and at the moment of transition from single support to double support when the swing leg lands as well as foot placement at the completion of the GI task. The findings will provide valuable insights for the clinical assessment of gait-related neurological disorders and the evaluation of the effectiveness of rehabilitation interventions in older people.

### Dynamic stability

GI involves internal self-perturbations of balance, known as endogenous ‘action equilibration’ (Bouisset and Do, 2008). MoS, derived from dynamic stability theory and the human inverted pendulum model, is a measure of walking stability by representing the shortest distance between a given CoM position–velocity state point and the backward imbalance boundary. Gait stability can be evaluated by calculating stability margins in the A-P and M-L directions, with the M-L dynamic MoS as a key indicator of walking stability.

The results show a statistically significant difference in M-L stability between SG and TC groups at the moment the swing leg is lifted off the ground during the transition from double-to single-leg support. M-L stability is an important predictor of fall risk, and the results suggest that sedentary older women are potentially at risk of falling. Nakano et al. also suggested that age-related changes in M-L balance control may increase the risk of lateral falls during GI (Nakano et al., 2016). Breniere and Do. (1986) reported that steady-state gait conditions are achieved at the end of the first step when walking at a habitual speed. The goal of GI is to place participants under steady-state gait conditions during the first step. At the end of the first step, the M-L and A-P stability in SG are significantly lower than those in TC, indicating that Tai Chi significantly improves the stepping gait stability during GI. At the onset of GI, sufficient APAs will ensure that the CoM is repositioned over the new support base (standing/supporting leg), preventing the body from tilting towards the swing leg after transitioning from bipedal to unipedal stance. If APAs fail to effectively reposition the CoM onto the new support surface, then walkers will need to rely on CPAs to reduce the effect of this instability, typically by taking more side steps with the stepping foot to increase the range of the support surface (Zettel et al., 2002). Some adaptation effects are consistent with the role of motor predictions in coupling posture and movement. The CNS anticipates the direction and magnitude of the initial lateral body movement, corresponding to the expected position of the M-L foot placement. Foot placement is adapted by altering posture and movement before stepping to ensure stability during stepping. These adaptations during movement may directly alter stepping performance. Disruptions in frontal plane postural control prompt movement adjustments to maintain stability and efficiency (e.g., maintaining step length) during the first step (Mille et al., 2007).

This study compares the displacement results of the CoP during GI at different angles (45° left anterior, 45° right anterior and 90° right lateral). A 16-week Tai Chi intervention significantly increases the displacement and speed of lateral steps in sedentary older adults. Tai Chi may improve dynamic postural control during the initial phase of GI in older adults by altering the movement characteristics of the CoP. Another study revealed that a 12-week Tai Chi exercise can improve posture control on unstable platforms and reduce the risk of falls in older individuals with sarcopenia (Huang et al., 2023). However, a previous research that compared the minimum postural sway ability of older adults suggested that a 15-week Tai Chi intervention did not improve measures of platform postural stability (Wolf et al., 1997). Our study includes stability calculations and reports that during the end stage of step advancement, the M-L and A-P stability of older women in the TC is significantly higher than that in sedentary older adults. Hence, Tai Chi exercise has a positive effect on improving the stability of GI in older adults. The different results of Tai Chi in improving postural stability may be due to the specific movement characteristics of Tai Chi, such as stable shifts in the centre of gravity (CoG) and slow, smooth transitions in step movements, which may manifest stability in controlling step movements.

### Foot placement

A potential method used to improve postural stability during GI is modifying the initial foot position (IFP). The human body achieves safe and efficient step advancement by adjusting the step length and position of the swing leg foot at ground contact. Considering the significance of appropriate IFP for GI and recovery response, the observed changes in step length during the first step of older fallers may serve as an important predictor of posture-related problems (Azizah Mbourou et al., 2003). During GI, coarse balance control is achieved through the placement of the swing leg’s foot (Jian et al., 1993). This study found statistically significant differences in the step length, width and speed of older females in the SG and TC group, with SG exhibiting significantly shorter step length and width. These findings align with previous research, which indicated that step length and step speed significantly decrease with age during GI (Muir et al., 2014) and that older fallers demonstrate significantly shorter first steps (Azizah Mbourou et al., 2003). Additionally, older adults can modulate their step width to maintain dynamic balance in the M-L direction (Nakano et al., 2016). Muir et al. (2014) compared age-related changes in the first four steps of gait, including the GI phase, for three age groups (20–25 years, 65–79 years and 80–91 years) walking at self-selected speeds. The results showed significant decreases in step speed and length across age groups. Higher variability in step length and step width may reflect increased foot placement errors and/or decreased CoG control among older adults. Healthy individuals aged 80–91 years walked at a slower pace with shorter steps. Furthermore, older fallers had significantly shorter first steps during GI and significantly longer double support time (Azizah Mbourou et al., 2003). Our results further confirm that older adults may utilise their foot placement at touchdown to regulate stable control at the end of the stance phase. A faster gait requires longer anticipation time and shorter execution time (Brenière et al., 1987). No statistically significant differences were observed in the foot inclination angle between the two groups. Inclination of the foot to the outer side (IFP) may be a feasible option to increase ankle muscle activation and improve age-related decline in postural stability (Jeon et al., 2021). Reinmann et al. (Reimann et al., 2017) proposed that foot placement and ankle lateral strategies may be two independent mechanisms that are coupled and coordinated in time to compensate for unstable M-L APAs and stiffened stance leg in the frontal plane to maintain body sagittal stability (Van Dieёn et al., 2008). The foot placement position is actively regulated by the CNS to maintain lateral balance (Bauby and Kuo, 2000; Donelan et al., 2004). The human body maintains gait stability by coordinating the M-L foot placement position and the control of the CoM (O’Connor and Kuo, 2009). The CNS anticipates the initial lateral displacement of the body and adjusts the foot placement accordingly to ensure its alignment with the expected position. The foot position is adapted by altering the pre-step posture to ensure stability during foot strikes (Collins and Kuo, 2013). Even if the foot placement position is slightly inappropriate at foot strike, it can still be compensated by adjusting the M-L position of the CoP, which is known as the ‘ankle lateral strategy’ (Hof et al., 2007). Changes in the length of the first step may be an important predictor of postural problems in older adults considering the importance of appropriate foot placement for GI and recovery responses.

### Strengths and limitations

This study compared the GI stability characteristics and foot placement features between two representative older groups, namely, sedentary older women and older women with long-term regular Tai Chi exercise. The results provide a reference for future studies on GI stability in specific population subgroups. However, this study did not investigate the coordination of limb spatial positioning and ground reaction forces. In future research, we will consider the coordination of GI and potential differences in force control that may lead to an increased risk of injury. Previous studies indicated that gender is a major risk factor for falls, with older women being more prone to falls. The present work only included older women participants and did not further categorize them by age group. Further studies will consider additional age groups and physical activity groups for research purposes.

## Conclusion

This study quantitatively compared and analysed the dynamic stability and foot placement characteristics of sedentary older women and older women with long-term regular Tai Chi exercise. The findings revealed that long-term regular Tai Chi exercise significantly improved the dynamic stability at the swing leg toe-off moment in M-L directions and at the heel–strike moment in the M-L and A-P directions, moreover, Tai Chi exercisers showed significantly higher step length, step width and step velocity than sedentary older women during GI. The results further confirm the negative effect of sedentary behaviour on stability control among older adults as well as the positive effects of Tai Chi on improving gait stability control and reducing fall risk.

## Data Availability

The original contributions presented in the study are included in the article/Supplementary Material, further inquiries can be directed to the corresponding authors.

## Abbreviations

GI, gait initiation; SG, sedentary group; TC, Tai Chi group; CoM, centre of mass; CoP, centre of pressure; CoG, centre of gravity; APAs, anticipatory postural adjustments; CPAs, compensatory postural adjustments; BMI, body mass index; MMSE, Mini-mental State Examination; BoS, base of support; MoS, margin of stability; M-L, medio-lateral; A-P, anterior-posterior; CNS, central nervous system; XcoM, extrapolated center of mass; SL, step length; SW, step width; SV, step velocity; SA, step inclination angle; IFP, initial foot position.
